# Spontaneous Cervical Spondylodiscitis With Retropharyngeal Abscess and Bacteremia: A Case Report

**DOI:** 10.7759/cureus.40246

**Published:** 2023-06-11

**Authors:** Lucie Cunha, Manuel Almeida, Isa Cordeiro, Alexandre Baptista

**Affiliations:** 1 Intensive Care Unit, Centro Hospitalar Universitário do Algarve - Portimão, Portimão, PRT

**Keywords:** immunosuppression, staphylococcus aureus, bacteremia, retropharyngeal abscess, cervical spondylodiscitis

## Abstract

Retropharyngeal abscess is a deep neck infection, rarely reported in adults. Nevertheless, when it occurs, it is mostly in immunocompromised patients and it can have life-threatening complications such as airway obstruction. On the other hand, more insidious complications can develop, such as mediastinitis, spinal osteomyelitis, and epidural abscess which represent an emergency medical condition when the patient develops neurologic symptoms. All must be diagnosed early and treated promptly. Spinal infection is an ancient disease, yet, morbidity remains significant despite developments in surgical and radiologic methods and the discovery of antibiotics. Management frequently involves a combination of these to achieve the best results.

The aim of this case report, as a research design, is to describe scientific observations that we encountered in a clinical setting, expand our knowledge, and highlight the role of the interprofessional team in evaluating and managing these conditions.

## Introduction

Infectious spondylodiscitis, an infection of the vertebral column, is a term encompassing vertebral osteomyelitis, spondylitis, and discitis. In patients over 50 years of age, it is the main manifestation of hematogenous osteomyelitis and accounts for 3%-5% of all cases of osteomyelitis [1].

There is a bimodal age distribution with peaks in the under 20-years-age group and in the group aged 50-to-70-year-olds, although all ages may be affected. Vertebral osteomyelitis is predominantly male, with a male-to-female ratio of 1.5-2:1. Risk factors for vertebral osteomyelitis include injection drug use, infective endocarditis, degenerative spine disease, prior spinal surgery, diabetes mellitus, corticosteroid therapy, or other immunocompromised states. The incidence is 7.4 per 100,000 and increasing due to an expansion in the susceptible population and improved diagnostic capabilities [2-3].

*Staphylococcus aureus* is the predominant pathogen, accounting for about half of non-tuberculous cases [4]. Diagnosis can be difficult since it is based on clinical, laboratory, and radiologic features. Because of the rarity of the disease, the insidious onset of symptoms, and the high incidence of back pain in the general population, it is often delayed or overlooked [5]. Patients present with persistent low back pain, fever, or neurologic findings. MRI has high sensitivity and specificity in diagnosing and differentiating the type of spondylodiscitis and can reveal signs of spondylodiscitis at very early stages [1].

The goal of treatment is to eliminate the infection, restore, and maintain the structure and function of the spine, and relieve pain. Conservative management, with non-pharmacological treatments such as physiotherapy and immobilization, is imperative. Still, infectious spondylodiscitis responds to antimicrobial therapy well if diagnosed early before the development of neurological deficit and the requirement of surgical intervention [6].

We present a clinical case of spondylodiscitis that developed in a young man with rheumatoid arthritis and a history of post-traumatic chronic osteomyelitis in the lower limb.

## Case presentation

The authors present a clinical case of a male adult, aged 59 years old. For the management of rheumatoid arthritis, he was medicated with Deflazacort 30 mg once daily for the past 3 years and denied taking other disease-modifying antirheumatic drugs. With the exception of weight gain after the initiation of chronic corticosteroid therapy, the patient denied other side effects associated with the treatment. He also had post-traumatic chronic osteomyelitis in the left tibia over 20 years ago and had a follow-up with orthopedics.

At admission to the hospital, he complained of lower back pain, limitation of the neck range, numbness, and weakness in both arms for 1 week. The patient denied a history of recent trauma (either to the lower limb or spine). There was a fever, tachycardia, and polypnea. Physical examination revealed hyperemia in the oropharynx and tenderness. Left lower limb with deformity in the anterior medial region, the target of multiple surgeries, but without apparent loss of skin continuity. In addition, neurological examination revealed dysphagia, ataxia, and dysmetria.

The peripheral white blood cell count was 23800/mm3 and C-reactive protein was elevated to 337 mg/L. Blood lactate was under 2 mmol/L and both renal and liver functions were normal. Human immunodeficiency virus and tuberculosis skin and blood tests were negative.

**Figure 1:**
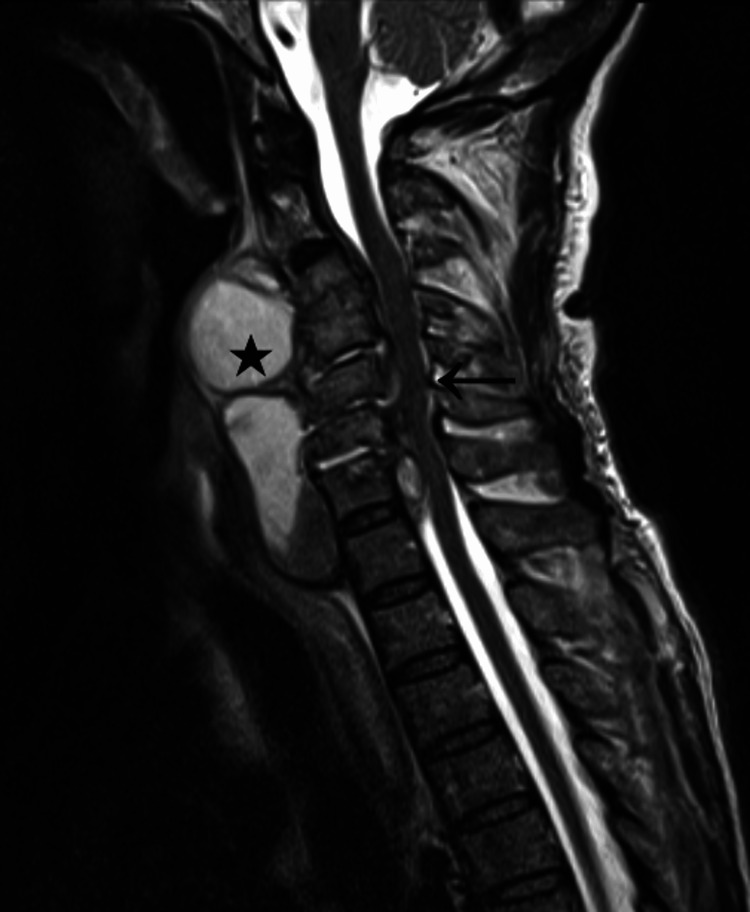
Sagittal section of the spine on MRI: the star shows the retropharyngeal abscess and the arrow shows the formation of the spinal cord.

 A cervical contrast-enhanced CT revealed retropharyngeal abscess; grade I retrolisthesis of C3, C5, and C6; frank reduction of the intersomatic space C3-C4; soft tissue swelling at the pre-vertebral level in the C3-D1 segment; posterior disc protrusions in C3-C7 that contact the medullary cord; decreases in foraminal permeability C3-C7, with possible root conflict; lytic foci of the vertebral bodies from C2 to C6. A cervical vertebrae MRI was then performed and showed pre-vertebral and extra-dural multiseptate collections, stenosing the cervical vertebral canal and with repercussions on the pharyngeal, inferior retroglossal, and hypoglossic airway, with subarachnoid effacement and slight molding of the spinal cord from C3 to C7 (Figure 1).

Several diagnosis hypotheses such as epiglottitis, severe tonsillopharyngitis, spinal epidural abscess, degenerative spine disease, herniated disc, and metastatic tumor were considered -- infectious spondylodiscitis and retropharyngeal abscess being the most probable. Empiric treatment with Ceftriaxone 2 g daily was started and maintained for 4 days, but methicillin-sensitive Staphylococcus aureus reproduced in the blood cultures taken at hospital admission, and antibiotic therapy with Flucloxacillin 12 g daily was instituted and preserved for 8 weeks. Also, the retropharyngeal abscess was drained surgically and the patient remained intubated after the surgery because of edema of the airway. There was no complication at the intraoperative or postoperative period and the patient was extubated 7 days later, after ruling out infectious complications, such as mediastinitis, and after evaluation by otolaryngology. Neck stabilization with a cervical collar was recommended and a more conservative approach was preferred by neurosurgery, with close monitoring given the risk of sudden neurological deterioration. Physical therapy was recommended to the patient.

**Figure 2:**
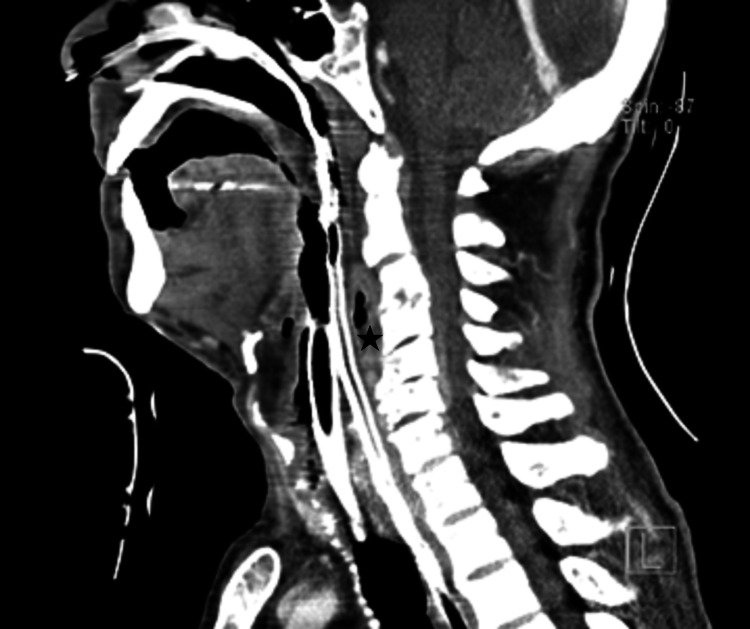
Postoperative cervical CT: the star shows the reduction of the retropharyngeal collection.

A good clinical and radiological evolution was observed. Cervical CT shows retropharyngeal collection measuring 20 mm x 8 mm in axial diameters in the C3 plane, with essentially air content with a post-drainage residual appearance; slightly more distal retroesophageal prevertebral collection, 7 mm thick, and the largest paravertebral collection in the upper right mediastinum, with a globose, multiseptated appearance, 22 mm thick; more evident spondylodysarthrosis alterations in C4-C6 and an erosive aspect in some vertebral platforms (Figure 2).

## Discussion

Since back pain is a nonspecific symptom in adults, this case reported was a diagnostic challenge, especially in a patient with a history of rheumatoid arthritis. Cervical pain and neurological sign could mean atlantoaxial instability, cranial settling, or subaxial subluxation due to rheumatoid arthritis complications. Post-traumatic chronic osteomyelitis in the patient's lower extremity likely contributed to the spinal infection and caused a leak in the barrier that promoted translocation of the pathogen to the circulatory system, leading to secondary infection of the spine. Suspicion of spondylodiscitis arose from physical signs such as fever and neurologic impairment. In addition, risk factors such as corticosteroid use, age > 50 years, and male sex were present.

Non-traumatic retropharyngeal abscesses and cervical spondylodiscitis are extremely rare in adults. However, because of the fatal and morbid complications, they are very important because they can cause permanent neurologic deficits or airway obstruction due to their proximity to some structures [2]. MRI should be the first imaging modality used in patients with suspected vertebral osteomyelitis. It has a sensitivity of 97%, a specificity of 93%, and an accuracy of 94%. The excellent morphologic resolution allows for the early detection of spondylitis.

The treatment strategy consists of both medical therapy and surgery. Drainage of the abscess and stabilization of the cervical spine are most commonly used. The use of long-term antimicrobial therapy is also required, depending on the culture results [1]. This patient was discharged 83 days later to convalescence. At this time, he had cachexia and dysphagia requiring a nasogastric tube, with no other neurological disorders. He was also advised to wear a neck brace and infectious diseases, orthopedics, and neurosurgery consultation were scheduled.

## Conclusions

Vertebral osteomyelitis is a very uncommon cause of concurrence with retropharyngeal abscess. Nevertheless, the fatal and morbid complications make it exceptionally important. Vertebral osteomyelitis and retropharyngeal abscess should be considered not only in patients with obvious risk factors. Their incidence is growing due to an increasingly susceptible population, the availability of more effective diagnostic tools, and the awareness of medical staff. Early diagnosis is important, and a high index of suspicion is required to make a rapid diagnosis and choose appropriate therapeutic agents. Microbiological diagnosis is essential to allow effective treatment. However, they must be considered medical-surgical emergencies, as they are likely to lead to serious complications.

To avoid complications and delays, a coordinated approach consisting of an otolaryngologist, a neurosurgeon, an intensivist, and an infectious disease specialist was required. The core function of a multidisciplinary team was to bring together a group of healthcare professionals from different fields in order to determine patients' invasive or conservative treatment plan and guarantee safety in the post-surgery period if needed. Advances in diagnostic and therapeutic procedures, as well as advances in critical care, have been instrumental in improving the prognosis and mortality of these patients.
